# Anti-Inflammatory Azaphilones from the Edible Alga-Derived Fungus *Penicillium sclerotiorum*

**DOI:** 10.3390/md19100529

**Published:** 2021-09-22

**Authors:** Hui-Chun Wang, Tzu-Yi Ke, Ya-Chen Ko, Jue-Jun Lin, Jui-Sheng Chang, Yuan-Bin Cheng

**Affiliations:** 1 Graduate Institute of Natural Products, College of Pharmacy, Kaohsiung Medical University, Kaohsiung 807378, Taiwan; wanghc@kmu.edu.tw (H.-C.W.); cj96u04@gmail.com (T.-Y.K.); helly06070@gmail.com (Y.-C.K.); 2 Department of Marine Biotechnology and Resources, National Sun Yat-sen University, Kaohsiung 804351, Taiwan; conk4wu0g3@gmail.com; 3 Department of Medical Research Center, Kaohsiung Medical University Hospital, Kaohsiung 807378, Taiwan; 4 Center of Excellence for the Oceans, National Taiwan Ocean University, Keelung City 202301, Taiwan; jschang@mail.ntou.edu.tw; 5 Department of Fragrance and Cosmetic Science, College of Pharmacy, Kaohsiung Medical University, Kaohsiung 807378, Taiwan; 6 Doctoral Degree Program in Marine Biotechnology, National Sun Yat-sen University, Kaohsiung 804351, Taiwan

**Keywords:** *Penicillium sclerotiorum*, azaphilone, anti-inflammatory

## Abstract

To discover the new medical entity from edible marine algae, our continuously natural product investigation focused on endophytes from marine macroalgae *Grateloupia* sp. Two new azaphilones, 8a-*epi*-hypocrellone A (**1**), 8a-*epi*-eupenicilazaphilone C (**2**), together with five known azaphilones, hypocrellone A (**3**), eupenicilazaphilone C (**4**), ((1*E*,3*E*)-3,5-dimethylhepta-1,3-dien-1-yl)-2,4-dihydroxy-3-methylbenzaldehyde (**5**), sclerotiorin (**6**), and isochromophilone IV (**7**) were isolated from the alga-derived fungus *Penicillium sclerotiorum*. The structures of isolated azaphilones (**1**–**7**) were elucidated by spectrometric identification, especially HRESIMS, CD, and NMR data analyses. Concerning bioactivity, cytotoxic, anti-inflammatory, and anti-fibrosis activities of those isolates were evaluated. As a result, compound **1** showed selective toxicity toward neuroblastoma cell line SH-SY5Y among seven cancer and one fibroblast cell lines. 20 μM of compounds **1**, **3**, and **7** inhibited the TNF-α-induced NFκB phosphorylation but did not change the NFκB activity. Compounds **2** and **6** respectively promoted and inhibited SMAD-mediated transcriptional activities stimulated by TGF-β.

## 1. Introduction

Edible marine algae, such as Japanese kelp, *Eucheuma* seaweeds, and *Gracilaria* seaweeds, are believed to have diverse pharmacologic functions and are regarded as valuable food sources for human beings. In 2018, 32.4 million tonnes of aquatic algae were produced and consumed worldwide [1]. Although farming aquatic algae is relatively environmentally friendly, many researchers still pursue a rapid and effective way to obtain those wholesome ingredients. Recently, a few papers reported that endophytes isolated from marine algae could be potential sources of novel natural products [2,3]. Therefore, purifying seaweed symbiotic bacteria and using their ingredients as new drug leads became important research topics.

The fungus of *Penicillium*, belonging to the family Trichocomaceae, is an important genus for drug production. More than 200 compounds isolated from marine-derived *Penicillium* species have been found to have strong cytotoxic or anti-tumor effects [4]. The natural product studies of *P. sclerotiorum* have led to the identification of a series of azaphilones [5,6,7], isocoumarin [8], and diterpenoids [9]. Some of those azaphilones showed anti-inflammatory [5], antimicrobial [8], antiviral [10], and cytotoxic [7] activities.

By coincidence of the coronavirus disease 2019 (COVID-19) pandemic, rapid mass observations from clinical revealed the feature of severe acute respiratory syndrome coronavirus 2 (SARS-CoV-2) infection. Accumulated evidence indicates that severe cytokine storms result in acute respiratory distress syndrome (ARDS) related lung tissue damage is the leading cause of death of patients infected with the coronavirus [11]. Cytokine storms meaning a considerable amount of cytokines and chemokines released by infiltrating immune cells in insulted tissue. Among them, proinflammation cytokines tumor necrosis factor (TNF-α) mediates NFκB downstream signal transduction is of notice. Neutralization therapeutics of TNF-α are suggested to managing the cytokine storm [12]. Pulmonary fibrosis is a known pathologic sequel to ARDS. The multifunctional cytokine TGF-β is one of the critical factors that contribute to tissue fibrosis. The canonical profibrotic pathway mediated by TGF-β/SMADs is also a significant target for anti-fibrotic therapies [13]. Therefore, developing agents against inflammation as well as the subsequent fibrosis processes are critical needs for preventing such irreversible tissue damage. To this end, we tested azaphilone compounds in TNF-α-induced NFκB activation and TGF-β-induced Smad activation. To our knowledge, the azaphilone compounds have never been evaluated by both pathways.

## 2. Results

**Figure 1:**
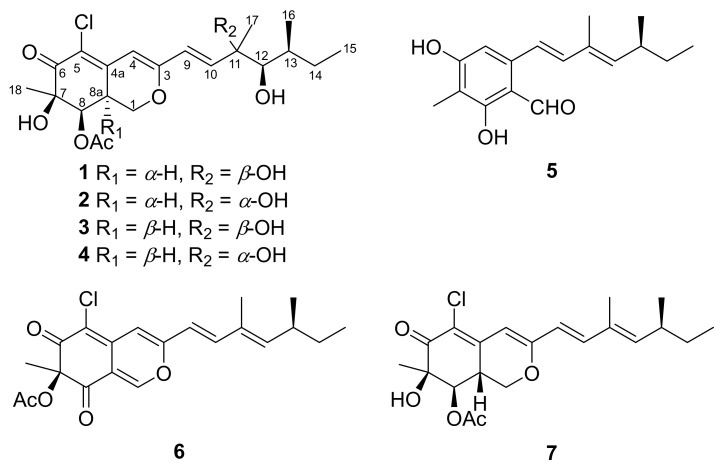
Structures of compounds **1**–**7** isolated from *Penicillium sclerotiorum*.

Meticulous chromatography of the extracts of *Penicillium sclerotiorum* resulted in the isolation of two new (**1** and **2**) and five known (**3**–**7**) azaphilones. Those isolated compounds were identified as 8a-*epi*-hypocrellone A (**1**), 8a-*epi*-eupenicilazaphilone C (**2**), together with five known azaphilones, hypocrellone A (**3**) [14], eupenicilazaphilone C (**4**) [14], ((1*E*,3*E*)-3,5-dimethylhepta-1,3-dien-1-yl)-2,4-dihydroxy-3-methylbenzaldehyde (**5**) [15], sclerotiorin (**6**) [16], and isochromophilone IV (**7**) [17] (Figure 1).

### 2.1. Structure Elucidation of New Compounds ***1*** and ***2***

**Figure 2:**
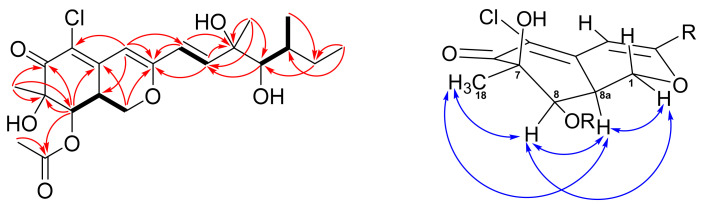
COSY (bold bond), selected HMBC (red arrows), and NOESY (left-right arrows) correlations of **1**.

According to the HRESIMS data (*m*/*z* 451.1493, [M+Na]^+^), a molecular formula of C_21_H_29_ClO_7_ was assigned to **1**. The UV absorption maxima at λmax 351 and 250 nm, and the IR absorption bands at 3472 (OH), 1708, and 1677 (carbonyls) cm^−1^, implied that **1** possesses an azaphilone skeleton. The ^1^H NMR spectrum of **1** (Table 1) exhibited a *trans* C=C double bond at δ 6.30 (d, *J* = 15.5; H-9) and δ 6.55 (d, *J* = 15.5; H-10), an olefinic singlet at δ 6.10 (H-4), two oxymethines at δ 5.52 (d, *J* = 2.5; H-8) and δ 3.49 (brs, H-12), an oxygen-bearing methylene at δ 4.47 (dd, *J* = 10.8, 4.8; H-1*α*) and δ 3.81 (m, H-1*β*), two methines at δ 3.23 (m; H-8a) and δ 1.70 (m, H-13), an acetate methyl at δ 2.02 (H-20), and four methyls at δ 0.92 (t, *J* = 7.0; H-15), δ 0.97 (d, *J* = 6.8; H-16), δ 1.32 (s; H-17), and δ 1.45 (s; H-18). Inspection of the ^13^C and DEPT data of **1** (Table 1) demonstrated twenty-one carbons of an azaphilone, including one conjugated carbonyl (δ 192.5), one acetyl group (δ 170.7 and 20.7), three olefinic quaternary carbons (δ 117.4, 144.3, and 161.0), three olefinic methines (δ 102.6, 112.6, and 144.5), two oxymethines (δ 74.4 and 78.5), two oxygen-bearing quaternary carbons (δ 76.0 and 76.3), one oxymethylene (δ 67.5), two methines (δ 35.5 and 36.9), one aliphatic methylene (δ 28.8), and four methyls (δ 12.0, 13.5, 23.7, and 24.6). The 6/6-fused bicyclic ring moiety of azaphilone was established by COSY correlation of H-1/H-8a/H-8 and HMBC correlations of H-1/C-3 (δ 161.0), C-4a (δ 144.3), H-4/C-3, C-5 (δ 117.4), and H-8/C-4a, C-6 (δ 192.5), C-7 (δ 76.3) (Figure 2). The HMBC correlations from Me-18 to C-6, C-7, C-8 indicated a methyl and a hydroxy group attaching at C-7. In addition, the HMBC correlations from both H-8 and H-20 to C-19 revealed an acetyl group attaching at C-8. The COSY spin systems of H-9/H-10 and H-12/H-13/Me-16, together with the HMBC correlations from H-15 to C-13 (δ 67.5) and C-14 (δ 67.5), from H-17 to C-10 (δ 141.6), C-11 (δ 76.2), C-12 (δ 79.2), and from H-9 to C-3 were used to construct the side chain of azaphilone with two hydroxy groups located at C-11 and C-12. Finally, a chloride atom was assigned at C-5 by virtue of the MS analysis (*m*/*z* 451 and 453 showed a ratio of 3:1) and the characteristic carbon chemical shift [14]. Therefore, the planar structure of compound **1** was established.

**Figure 3:**
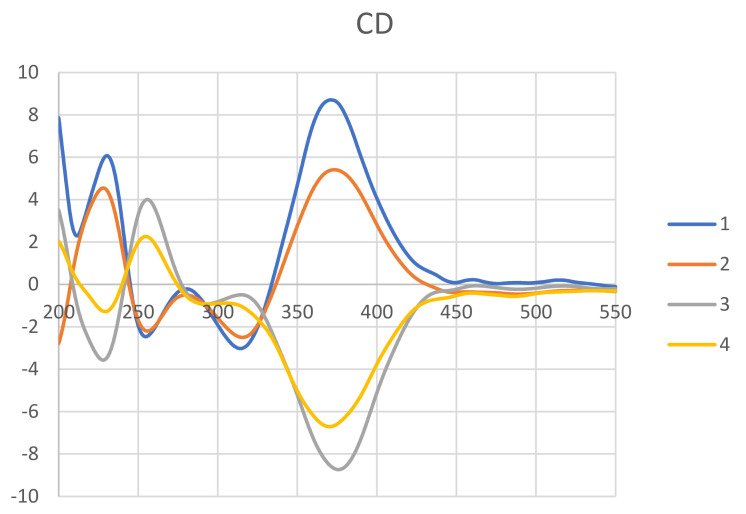
Experimental ECD spectra of **1**−**4**.

The absolute stereochemistry of **1** was determined by the NOESY experiment along with 1D NMR data and CD spectra comparison. The NOESY correlations of Me-18/H-8/H-8a/H-1α (Figure 2) revealed those protons located on the same face. Thus, the configuration of C-8a was suggested to be *S*, which was opposite to compound **3**. The chiral centers of the side chain (C-9 to C-17) were determined by comparing the ^1^H and ^13^C data between **1** and **3** (Table S1). The Me-17 of **1** and **3** showed a characteristic proton value around δ 1.32 and carbon value at δ 23.7. In addition, the H-12, H-13, and Me-16 chemical shifts of **1** consisted of those of **3**. Therefore, the configurations of the side chain were assigned to be 11*R*,12*R*,13*S*. To confirm the above assignment, CD experiments of compounds **1**–**4** were carried out. The positive cotton effect at 371 nm of **1** and the negative cotton effect at 375 of **3** (Figure 3) suggested the C-8a configuration of these two azaphilones was revered. On the basis of the above spectroscopic data analyses, the structure of **1** was assigned unambiguously and named 8a-*epi*-hypocrellone A.

**Figure 4:**
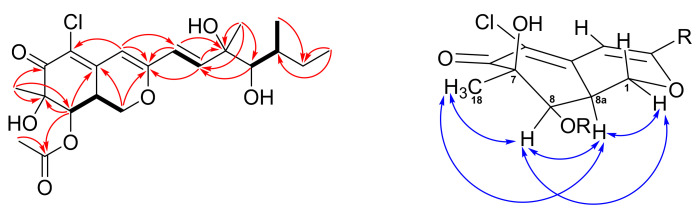
COSY (bold bond), selected HMBC (arrow), and NOESY (left-right arrow) correlations of **2**.

New compound **2** was isolated as pale yellowish amorphous gum. Its HRESIMS data showed a sodiated ion peak at m/z 451.14912, which agrees with the molecular formula of C_21_H_29_ClO_7_. The almost identical ^1^H, ^13^C, COSY, and HMBC NMR spectrometric data (Figure 4) between **2** and eupenicilazaphilone C (**4**) suggested their structure must be quite similar. Their main differences in the ^1^H NMR spectrum are the chemical shifts of H-8 (δ 5.52 for **2** and δ 5.00 for **4**) and H-8a (δ 3.25 for **2** and δ 3.45 for **4**), that implied that **2** could be a stereoisomer of **4**. The NOESY correlations of Me-18 (δ 1.45)/H-8 (δ 5.52)/H-8a (δ 3.25)/H-1α (δ 4.49) (Figure 4) indicated that H-8a should be α-oriented (8a*S*), which is the same as **1** but opposite to **4**. The chemical shifts of C-11 to C-13 between **2** and **4** were very close (Table S1), suggesting the same configuration (11*S*,12*R*,13*S*). The CD data of **2** also demonstrated a similar trend as that of **1**, indicated the 7*R*,8*R*,8a*S* configuration of those chiral centers. Therefore, the structure of 8a-*epi*-eupenicilazaphilone C (**2**) was defined as shown.

### 2.2. Cytotoxicity of Compounds ***1***–***7***

Compounds **1**–**7** were tested for their anti-cancer activity on lung cancer cells of A549 and CL1-5, breast cancer cells of MCF-7 and MDA-MB-231, colon cancer cells of HCT15 and HCT116, neuroblastoma cells SH-SY5Y, and normal lung fibroblast cells WI-38. When compounds were treated in cells with a general density of 1 × 10^4^/96-well, compounds **1**–**7** showed no cytotoxicity with the IC_50_ greater than 100 μM in most cells, in comparison with doxorubicin with an IC_50_ range from 0.36–3.7 μM in various cancer cells (Table 2). Only if compounds were treated in cells with a very low density of 1 × 10^3^/96-well can the tested concentration achieve a half-inhibition. We consider **1**–**7** are relatively less toxic compounds (Table 3). It is noteworthy that compound 1 showed selective cytotoxicity on neuroblastoma cells SH-SY5Y with an IC_50_ of 26.8 and 35.6 μM in different confluence.

### 2.3. Anti-Inflammatory Activity of Compounds ***1***–***7***

**Figure 5:**
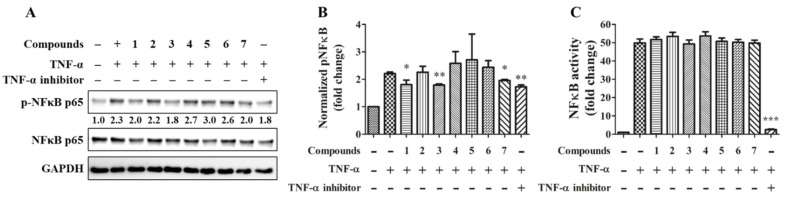
Effects of compounds on NFκB signaling and activity. 20 ng/mL TNF-α together with or without 20 μM of the individual compound were treated to TPH cells for 30 min (**A**,**B**; *n* = 3) or to the NFκB reporter cells for 24 h (**C**; *n* = 6). 10 μM IκB/IKK inhibitor BAY 11-7082 serve as a positive control. * for *p* < 0.05, ** for *p* < 0.01, and *** for *p* < 0.001.

TNF-α is considered a critical cytokine in COVID-19–associated cytokine storm of acute inflammation, inflammatory bowel disease of chronic inflammation, and rheumatoid arthritis in autoimmune disease [18]. In order to test compounds with a potential effect on preventing inflammation from deteriorating, the TNF-α-induced NFκB phosphorylation was tested by immunoblotting (Figure 5A). The quantitative result showed that compounds **1**, **3**, **7**, and the NFκB inhibitor significantly reduce NFκB phosphorylation (Figure 5B). However, except for the NFκB inhibitor, the isolated compounds did not alter NFκB transcriptional activity by reporter assay (Figure 5C).

### 2.4. Anti-Fibrotic Activity of Compounds ***1***–***7***

**Figure 6:**
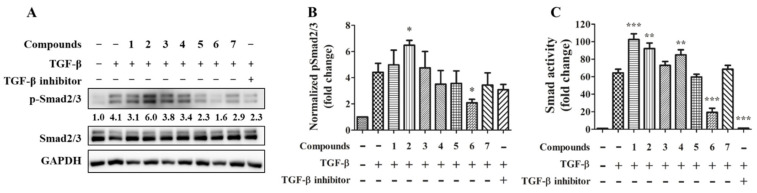
Effects of compounds on Smad2/3 signaling and activity. 20 ng/mL TGF-β together with or without 20 μM of the individual compound were treated to TPH cells for 30 min (**A** and **B**; *n* = 3) or to the SMAD reporter cells for 24 h (**C**; *n* = 6). 1 μM TGFβRI inhibitor LY3200882 (Figure 7) serve as a positive control. * for *p* < 0.05, ** for *p* < 0.01, and *** for *p* < 0.001.

Cytokine TGF-β is widely implicated in the pathogenesis of fibrosis in tissue organs. It has been proposed that effectively target TGF-β driving cell differentiation and synthesis or stabilization of extracellular matrix (ECM) can suppress fibrotic disease progression [19]. In order to test compounds with a potential effect on preventing tissue fibrosis, the TGF-β-induced Smad2/3 phosphorylation was tested by immunoblotting (Figure 6A). The quantitative result showed that compound transcriptional activity was tested. The result showed compounds **2** and **3** significantly increase and decrease Smad2/3 phosphorylation respectively (Figure 6B); the TGFβRI inhibitor showing a trend toward a decrease in Smad2/3 phosphorylation but approaching borderline statistical significance (*p* = 0.08). TGF-β stimulates SMAD transcriptional increased by compounds **1**, **2**, and **4** and decreased by compound **6** and the TGFβRI inhibitor (Figure 6C).

## 3. Discussion

Although the mechanism for selective cytotoxicity of compound **1** toward SH-SY5Y cells remains undetermined, we consider the coexistence of R = *α*-H and R1 = *β*-OH is favoring the selectivity by the comparison within compounds **1**–**4**.

**Figure 7:**
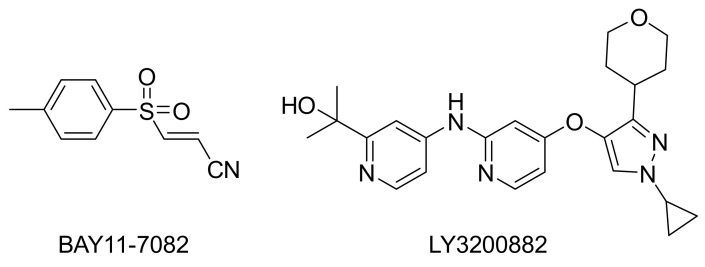
Chemical structures of references products BAY11-7082 and LY3200882.

Activation of NF-κB is an important event in TNF-α signaling. The phosphorylation of NFκB p65 Ser536 is regulated directly by IKK and is a marker of NF-κB activation. However, the NFκB activity represents its transcriptional function and is regulated by a complex network at least in part involved in GSK3 [20]. By immunoblot assay, we found compounds **1**, **3**, **7** inhibit NFκB phosphorylation at 30 min after TNF-α stimulation indicates TNF-α/TNF receptor/IKK axis is impeded, but the ultimate NF-κB transcriptional activity at 24 h remains by the observation in the reporter assay. BAY11-7082 (Figure 7) is an IKK kinase inhibitor that irreversibly inhibits phosphorylation and degradation of IkBα, thereby releasing p65/p50 heterodimer to translocate into the nucleus. BAY11-7082 also processes a broad spectrum inhibition on inflammatory signaling enzymes, such as Jak, PI3K, PDK1, and Akt [21]. We suggest the mechanism of action is the difference between azaphilone compounds and BAY 11-7082. The specificity of azaphilone compounds on IKK needs to be determined.

On the other hand, the result from immunoblot and transcription activity is similar in the investigation of the TGF-β/Smad pathway. Compound **2** could promote both TGF-β/Smad signaling and transcriptional function. Compound **6**, similar to the selective TGFβRI kinase inhibitor LY3200882, inhibited both TGF-β/Smad-mediated signaling and transcriptional function. The receptor TGFβRI kinase directly phosphorylates and activates Smad2/3, and it is fascinating to exam whether **6** has a specific effect on TGFβRI kinase in the future.

## 4. Materials and Methods

### 4.1. General

Sephadex (GE healthcare) LH-20 and silica gel 60 (Merck KGaA, Darmstadt, Germany) were used for open-column chromatography (CC). Luna C_18_, phenyl-hexyl (5 μm, 250 × 10 mm, Phenomenex, Torrance, CA, USA) semi-preparative columns were used for high-performance liquid chromatography (HPLC). HPLC instrument used a Shimadzu LC-10AT pump with an SPD-20A UV-Vis detector. The UV spectra were obtained by using a Jasco UV-530 ultraviolet spectrophotometer (Jasco, Tokyo, Japan), whereas the IR spectra were obtained on a Jasco FT-IR-4600 spectrophotometer (Jasco, Tokyo, Japan). Optical rotations were measured with a Jasco P-1020 digital polarimeter (Jasco, Tokyo, Japan). NMR spectra were obtained using JEOL JNM ECS 400 MHz (JEOL, Tokyo, Japan), Varian 600 MHz NMR (Varian, Palo Alto, CA, USA), and Bruker AVIIIHD700X 700 MHz spectrometers (Bruker, Bremen, Germany). ESI–MS data were collected on a VG Biotech Quattro 5022 mass spectrometer (VG Biotech, Altrincham, UK). High-resolution ESI–MS data were obtained with a Bruker APEX II spectrometer (Bruker, Bremen, Germany). Circular dichroism spectra were recorded on a JASCO J-810 spectrophotometer (Jasco, Tokyo, Japan).

### 4.2. Alga Material

The alga material was collected in May 2019 off the coast of Yilan County, Taiwan. Alga specimen was identified as *Grateloupia* sp. by co-author Dr. Jui-Sheng Chang. A voucher sample (specimen code: Al-27) was deposited at the Department of Marine Biotechnology and Resources, National Sun Yat-sen University, Kaohsiung, Taiwan.

### 4.3. Separation and Identification of Fungal Material

The alga material of *Grateloupia sp.* was soaked in 0.01% Tween 20 and treated with 0.01% bleach for surface cleaning. The disinfected alga specimen was cut in a size of about 5 mm × 5 mm. The sample was placed into the PDA (potato dextrose agar) medium and incubated at 25 °C. After continuous separation and purification of the hyphae, a pure fungal strain was obtained. The mycelium of pure fungal was lyophilized and ground. The DNA of powdered material (100 mg) was extracted using DNeasy Plant Mini Kit (Qiagen, Venlo, The Netherlands) following the manufacture’s protocol. Two sets of primers ITS4:′-TCCTCCGCTTATTGATATGC-3′/ITS5:5′-GGAAGTAAAAGTCGTAACAAGG-3′ were used to amplify the 18S rDNA. PCR amplifications were performed using KAPA HiFi DNA polymerase (Kapa Biosystems). The purified PCR products were analyzed by Genomic (New Taipei City, Taiwan). Reverse and forward results of nucleotide sequences of 18S r DNA were blasted using the National Center for Biotechnology Information database for species identification. The 18S rDNA gene sequence of the amplicon shares 100% sequence identity with *Penicillium sclerotiorum* (GenBank accession number: KM265451.1).

### 4.4. Extraction and Isolation

Fermentation broth of *P. sclerotiorum* (16.1 L) was partitioned between EtOAc and H_2_O, and the EtOAc extract (10.4 g) was further partitioned between hexanes and 75% MeOH to acquire a 75% MeOH-soluble extract. This extract (7.5 g) was subjected to a flash column eluted by stepwise hexanes/ethyl acetate/methanol (20:1:0~0:5:1) to obtain 15 fractions (PS1~PS15), according to TLC analysis. The second fraction (PS2, 44.0 mg) was isolated by reverse-phase HPLC (C_18_ column, 77% MeOH, isocratic), and compound **3** (1.1 mg) was obtained. Fraction PS4 (207.7 mg) was separated by a silica gel open column stepwise eluted with hexanes/dichloromethane/methanol (400:100:1~0:0:1) to get compound **5** (8.0 mg). Fraction PS6 (68.1 mg) was purified by reverse-phase HPLC (phenyl-hexyl column, 70% MeOH, isocratic) to afford compound **4** (11.6 mg). Fraction PS9 (291.5 mg) was isolated by a Sephadex LH-20 open column eluted with dichloromethane/methanol (1:1) to provide eight subfractions (PS9LH1~PS9LH8). Subfraction PS9LH4 (41.6 mg) was purified by reverse-phase HPLC (phenyl-hexyl column, 60% MeOH, isocratic) to yield compounds **1** (11.3 mg) and **7** (1.9 mg). Subfraction PS9LH5 (61.2 mg) was also separated by reverse-phase HPLC (C_18_ column, 48% MeOH, isocratic) to afford compounds **2** (8.2 mg) and **6** (19.1 mg).

### 4.5. Spectroscopic Data

8a-*epi*-hypocrellone A (**1**) pale yellowish amorphous gum, ${\left[\alpha \right]}_{D}^{27}$ +129 (*c* 0.05, MeOH); UV (MeOH) *λ*_max_ (log *ε*) 351 (4.00), 250 (3.25) nm; ECD (MeOH) *λ*_max_ (Δ*ε*): 231 (+6.07), 255 (−2.45), 314 (−3.02), 371 (+8.71) nm; IR (neat) ν_max_ 3472, 2961, 2925, 1708, 1678, 1566, 1373, 1234, 1151, 1092, 1043 cm^−1^; ^1^H-NMR (600 MHz) and ^13^C-NMR (150 MHz) see Table 1; HRESIMS *m/z* 451.14930 (calcd for C_21_H_29_ClNaO_7_, 451.14940).

8a-*epi*-eupenicilazaphilone C (**2**) pale yellowish amorphous gum, ${\left[\alpha \right]}_{D}^{27}$ +68 (*c* 0.05, MeOH); UV (MeOH) *λ*_max_ (log *ε*) 358 (3.80), 239 (3.22) nm; ECD (MeOH) *λ*_max_ (Δ*ε*): 228 (+4.55), 256 (−2.20), 316 (−2.49), 374 (+5.41) nm; IR (neat) *ν*_max_ 3481, 2958, 2925, 1742, 1674, 1568, 1373, 1231, 1151, 1100, 1040 cm^−1^; ^1^H-NMR (600 MHz) and ^13^C-NMR (150 MHz) see Table 1; HRESIMS *m/z* 451.14912 (calcd for C_21_H_29_ClNaO_7_, 451.14940).

### 4.6. Cytotoxicity Assays

The cell lines A549, MCF-7, MDA-MB-231, SH-SY5Y, and WI-38 were purchased from Bioresource Collection and Research Centre (BCRC; Taiwan). CL1-5 cells were gifted from Prof. Chin-Chung Wu (Kaohsiung Medical University, Taiwan), and HCT15 and HCT116 cells were given by Prof. Chen-Yang Shen (Academia Sinica, Taiwan). 1 × 10^3^ or 1 × 10^4^ cells were seeded in a 96-well one day before compounds treatment. Serially diluted concentrations for compounds from 100 μM and doxorubicin from 10 μM treated to cells for 48 h, cultured medium replaced by medium containing 0.5 mg/mL MTT (3-(4,5-dimethylthiazol-2-yl)2,5-diphenyl tetrazolium bromide) for additional 4 h of culture, the formazan formation was dissolved by 100 μL DMSO and quantified spectrophotometrically at 570 nm using an ELISA reader. The concentration of compound which exhibited 50% cell viability (IC_50_) was calculated by linear regression of the percentage survival versus the drug concentration.

### 4.7. Western Blot Assay

THP-1 monocyte cell line that was gifted from Prof. Tsung-Hsien Chang (National Defense Medical Center, Taiwan) was used to examine for TNF-α-induced NFκB phosphorylation; CCD966SK fibroblast cell line that purchased from Bioresource Collection and Research Centre (BCRC; Taiwan) was used to examine for TGF-β-induced Smad2/3 phosphorylation. The procedure of protein preparation, separation, blotting, and detection was conducted referred to previously [22]. The primary antibodies against phospho-NFκB p65(S536), NFκB p65, phospho-Smad 2(S465/467)/Smad 3(S423/425), and Smad 2/3 were purchased from Cell Signaling (USA). The level of Gapdh expression served as an internal control for protein loading. The annotated number represents the quantified level from normalized by both NFκB p65/Smad 2/3 and Gapdh and calculated the fold change phospho-NFκB or phospho-Smad 2/3 to their DMSO vehicle control group by ImageJ software. IκB/IKK inhibitor BAY 11-7082 (Cayman) and TGFβRI inhibitor LY3200882 (Medchemexpres) as positive controls.

### 4.8. NFκB and Smad Transcriptional Activity Assays

Reporter plasmids (Promega, Madison, WI, USA) with luciferase driven by NFκB or SMAD responsive elements were stably established in HEK293A cells and maintained by a puromycin-containing culture medium. Chemiluminescence levels represent the luciferase expression was determined by the ONE-Glo reagent (Promega, USA). The fold change of each group to the DMSO vehicle control group was calculated.

### 4.9. Statistics

Statistical analysis was conducted via a one-tailed unpaired *t*-test in comparison compound treating group with the group only treated with TNF-α or TGF-β by Microsoft Excel software. The significance was annotated on plots of the result, * for *p* < 0.05, ** for *p* < 0.01, and *** for *p* < 0.001.

## 5. Conclusions

*Penicillium sclerotiorum* was found to produce a wide structural diversity of azaphilones, which showed various bioactive effects, especially anti-inflammatory and cytotoxic properties. In this investigation of bioactive ingredients from marine resources, several edible macroalgae were collected around Taiwanese seashores, and an endophytic fungus, *P. sclerotiorum,* was successfully cultured in the PDA medium. Two new and five known azaphilones (**1**‒**7**) were identified from *P. sclerotiorum*, which agree with the secondary metabolite reported in the literature. The anti-inflammatory and anti-fibrotic ability of those isolates were assessed using NFκB and Smad transcriptional activity assays. The results showed azaphilones could be a new target for anti-inflammatory researches.

## Figures and Tables

**Table 1:** ^1^H-NMR and ^13^C-NMR data for compounds **1** and **2**.

	1 *^a^*		2 *^a^*	
	δ_H_ (*J* in Hz)	δc, Type	δ_H_ (*J* in Hz)	δc, Type
1*α*	4.47, dd (10.8, 4.8)	67.5, CH_2_	4.49, dd (10.9, 4.8)	67.6, CH_2_
1*β*	3.81, m		3.83, dd (13.1, 10.9)	
3		161.0, C		161.0, C
4	6.10, s	102.6, CH	6.11, s	102.6, CH
4a		144.3, C		144.3, C
5		117.4, C		117.3, C
6		192.5, C		192.5, C
7		76.3, C		76.2, C
8	5.52, d (2.5)	74.4, CH	5.52, d (3.1)	74.4, CH
8a	3.23, m	36.9, CH	3.25, m	36.9, CH
9	6.30, d (15.5)	112.6, CH	6.32, d (15.5)	112.2, CH
10	6.55, d (15.5)	144.5, CH	6.51, d (15.5)	141.6, CH
11		76.0, C		76.2, C
12	3.49, brs	78.5, CH	3.52, d (2.0)	79.2, CH
13	1.70, m	35.5, CH	1.55, m	36.6, CH
14	1.41, m	28.8, CH_2_	1.42, m	28.9, CH_2_
	1.33, m		1.31, m	
15	0.92, t (7.0)	12.0, CH_3_	0.91, t (7.4)	12.0, CH_3_
16	0.97,d (6.8)	13.5, CH_3_	0.88, d (7.0)	13.3, CH_3_
17	1.32, s	23.7, CH_3_	1.37, s	27.0, CH_3_
18	1.45, s	24.6, CH_3_	1.45, s	24.6, CH_3_
19		170.7, C		170.7, C
20	2.02, s	20.7, CH_3_	2.02, s	20.7, CH_3_

*^a^*^1^H and ^13^C-NMR were measured in CDCl_3_ at 600 and 150 MHz, respectively.

**Table 2:** Cytotoxicity of azaphilone compounds in a general density of cancer and fibroblast cells.

Compounds	A549	CL1-5	MCF-7	MDA-MB-231	HCT15	HCT116	SH-SY5Y	WI-38
**1**	>100	>100	>100	>100	>100	>100	35.6 ± 3.1	>100
**2**	>100	>100	>100	>100	>100	>100	73.2 ± 1.5	>100
**3**	>100	>100	>100	>100	>100	>100	>100	>100
**4**	>100	>100	>100	>100	>100	>100	95.2 ± 4.1	>100
**5**	>100	>100	>100	>100	87.2 ± 4.7	36.0 ± 0.8	>100	>100
**6**	>100	>100	>100	>100	>100	>100	>100	>100
**7**	>100	>100	>100	>100	>100	>100	>100	>100
doxorubicin	3.7 ± 0.1	3.6 ± 0.1	1.2 ± 0.1	3.4 ± 0.1	1.8 ± 0.5	0.5 ± 0.0	0.36 ± 0.0	>10

1 × 10^4^ per 96-well of cells were prepared for compound testing. The half-maximal inhibitory concentration (IC_50_; μg/mL) of cell survival was calculated using compound concentrations in serial dilution of 0, 6.25, 12.5, 25, 50, 100 μM/mL treated to cells for 48 h. Data represent Mean ± SEM from three independent experiments.

**Table 3:** Cytotoxicity of azaphilone compounds in a low density of cancer and fibroblast cells.

Compounds	A549	CL1-5	MCF-7	MDA-MB-231	HCT15	HCT116	SH-SY5Y	WI-38
**1**	>100	89.3 ± 4.9	>100	62.4 ± 4.0	88.2 ± 0.7	66.0 ± 3.8	26.8 ± 4.2	>100
**2**	>100	>100	>100	79.4 ± 0.6	94.2 ± 3.2	72.1 ± 2.5	>100	>100
**3**	>100	78.4 ± 5.0	68.8 ± 3.5	40.4 ± 2.8	76.1 ± 1.8	47.3 ± 2.0	>100	>100
**4**	>100	77.6 ± 3.0	80.4 ± 5.1	45.1 ± 0.8	76.9 ± 0.8	56.3 ± 0.6	>100	>100
**5**	44.3 ± 1.3	60.3 ± 3.8	52.1 ± 9.0	44.4 ± 0.6	78.6 ± 2.0	37.5 ± 0.8	>100	>100
**6**	>100	>100	>100	>100	>100	>100	>100	>100
**7**	>100	84.3 ± 4.3	92.8 ± 2.7	47.4 ± 1.2	61.0 ± 2.5	54.1 ± 2.0	>100	>100
doxorubicin	1.3 ± 0.1	2.2 ± 0.0	0.5 ± 0.0	0.5 ± 0.0	1.2 ± 0.3	0.38 ± 0.0	0.9 ± 0.3	>10

1 × 10^3^ per 96-well of cells were prepared for compound testing. The half-maximal inhibitory concentration (IC_50_; μg/mL) of cell survival was calculated using compound concentrations in serial dilution of 0, 6.25, 12.5, 25, 50, 100 μM/mL treated to cells for 48 h. Data represent Mean ± SEM from three independent experiments.

## Data Availability

Data available in a publicly accessible repository.

## Supplementary Materials

The ^1^H, ^13^C, HSQC, COSY, HMBC, NOESY, and HRESIMS spectra of compounds 1 and 2 are available online at www.mdpi.com/article/10.3390/md19100529/s1, Figure S1: The ^1^H-NMR spectrum of 1 (400 MHz in CDCl_3_), Figure S2: The ^13^C-NMR spectrum of 1 (100 MHz in CDCl_3_), Figure S3: The ^1^H-^1^H COSY spectrum of 1, Figure S4: The HSQC spectrum of 1, Figure S5: The HMBC spectrum of 1, Figure S6: The NOESY spectrum of 1, Figure S7: The ^1^H-NMR spectrum of 2 (400 MHz in CDCl_3_), Figure S8: The ^13^C-NMR spectrum of 2 (100 MHz in CDCl_3_), Figure S9: The 1H-1H COSY spectrum of 2, Figure S10: The HSQC spectrum of 2, Figure S11: The HMBC spectrum of 2, Figure S12: The NOESY spectrum of 2, Figure S13: The HRESIMS of 1, Figure S14: The HRESIMS of 2. Table S1 the ^1^H and ^13^C data of the sidechain of compounds 1–4.
