# Supplementary material for: Anti-Inflammatory Azaphilones from the Edible Alga-Derived Fungus Penicillium sclerotiorum
Source: Mar Drugs. 2021 Sep 22;19(10):529. doi: 10.3390/md19100529 (PMC8537458; doi:10.3390/md19100529)

Supporting Information

Anti-Inflammatory Azaphilones from the Edible Alga-Derived Fungus *Penicillium sclerotiorum*

Hui-Chun Wang, Tzu-Yi Ke, Ya-Chen Ko, Jue-Jun Lin, Jui-Sheng Chang, and Yuan-Bin Cheng*

Table of Contents

Figure S1 ^{1}H NMR spectrum of **1** ($CDCl_3$, 600 MHz)........2
Figure S2 ^{13}C NMR and DEPT spectrum of **1** ($CDCl_3$, 150 MHz).........3
Figure S3 COSY spectrum of **1**........4
Figure S4 HSQC spectrum of **1**........5
Figure S5 HMBC spectrum of **1**........6
Figure S6 NOESY spectrum of **1**........7
Figure S7 ^{1}H NMR spectrum of **2** ($CDCl_3$,600 MHz)........8
Figure S8 ^{13}C NMR and DEPT spectrum of **2** ($CDCl_3$, 150 MHz)........9
Figure S9 COSY spectrum of **2**........10
Figure S10 HSQC spectrum of **2**........11
Figure S11 HMBC spectrum of **2**........12
Figure S12 NOESY spectrum of **2**........13
Figure S13 HRESIMS spectrum of **1**........14
Figure S14 HRESIMS spectrum of **2**........15
Table S1 the ^{1}H and ^{13}C data of the sidechain of compounds **1**–**4**........16
Figure S15 IR spectrum of **1**........17
Figure S16 UV spectrum of **1**........17
Figure S17 IR spectrum of **2**........18
Figure S18 UV spectrum of **2**........18

Figure S1 ^1H NMR spectrum of **1** ($CDCl_3$, 600 MHz)

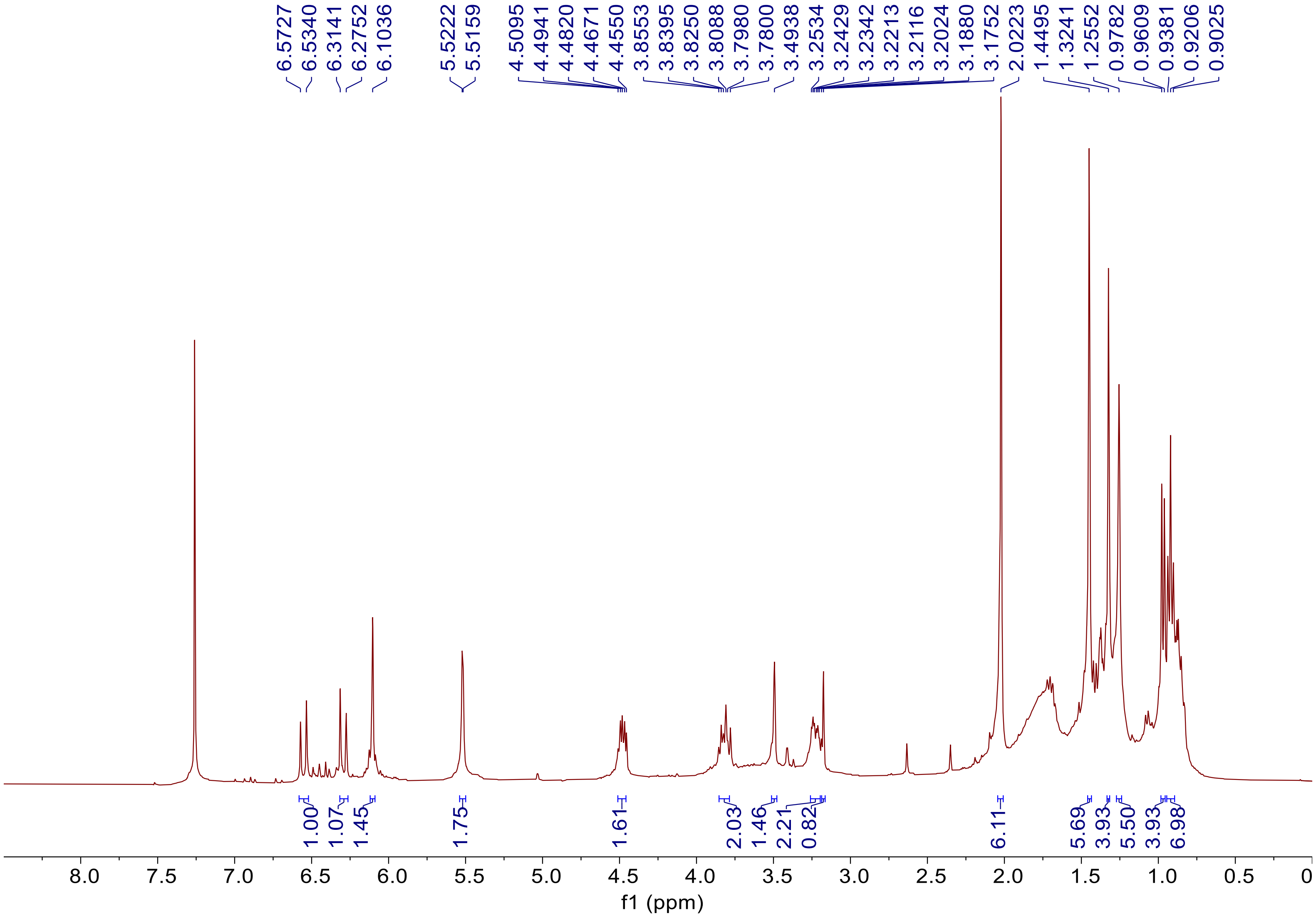

Figure S2 ^{13}C NMR and DEPT spectrum of **1** ($CDCl_3$, 150 MHz).

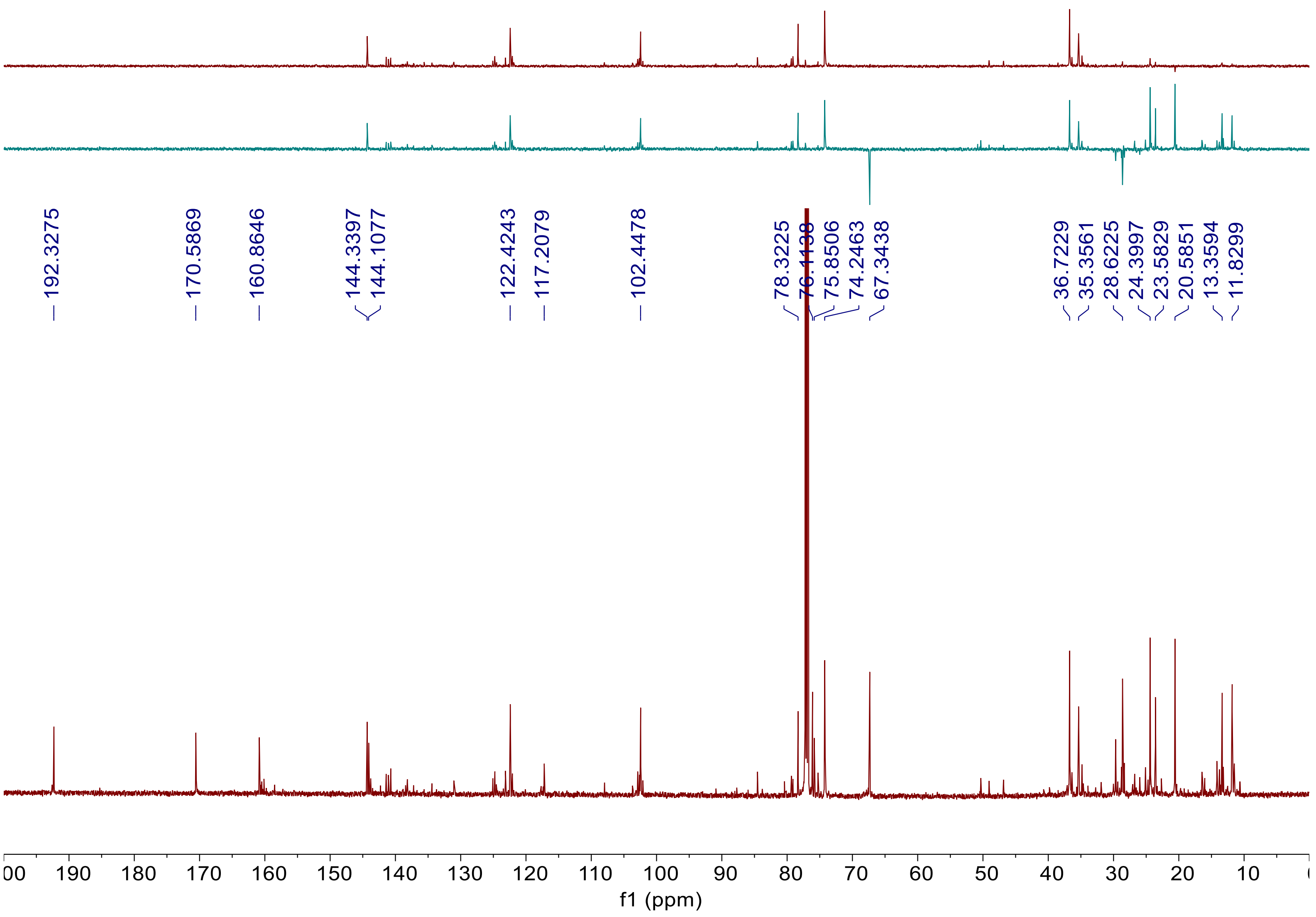

Figure S3 COSY spectrum of **1**

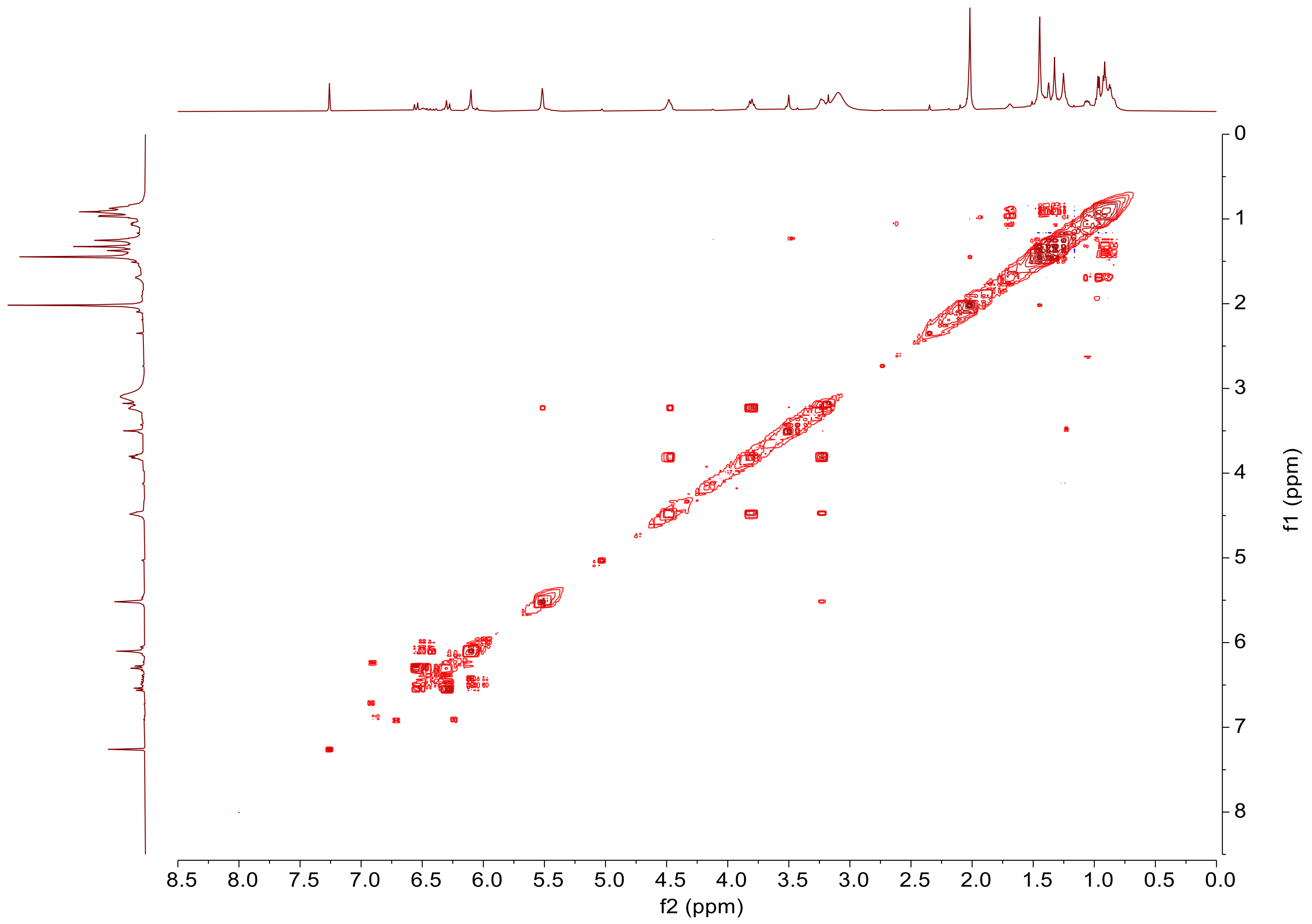

Figure S4 HSQC spectrum of **1**

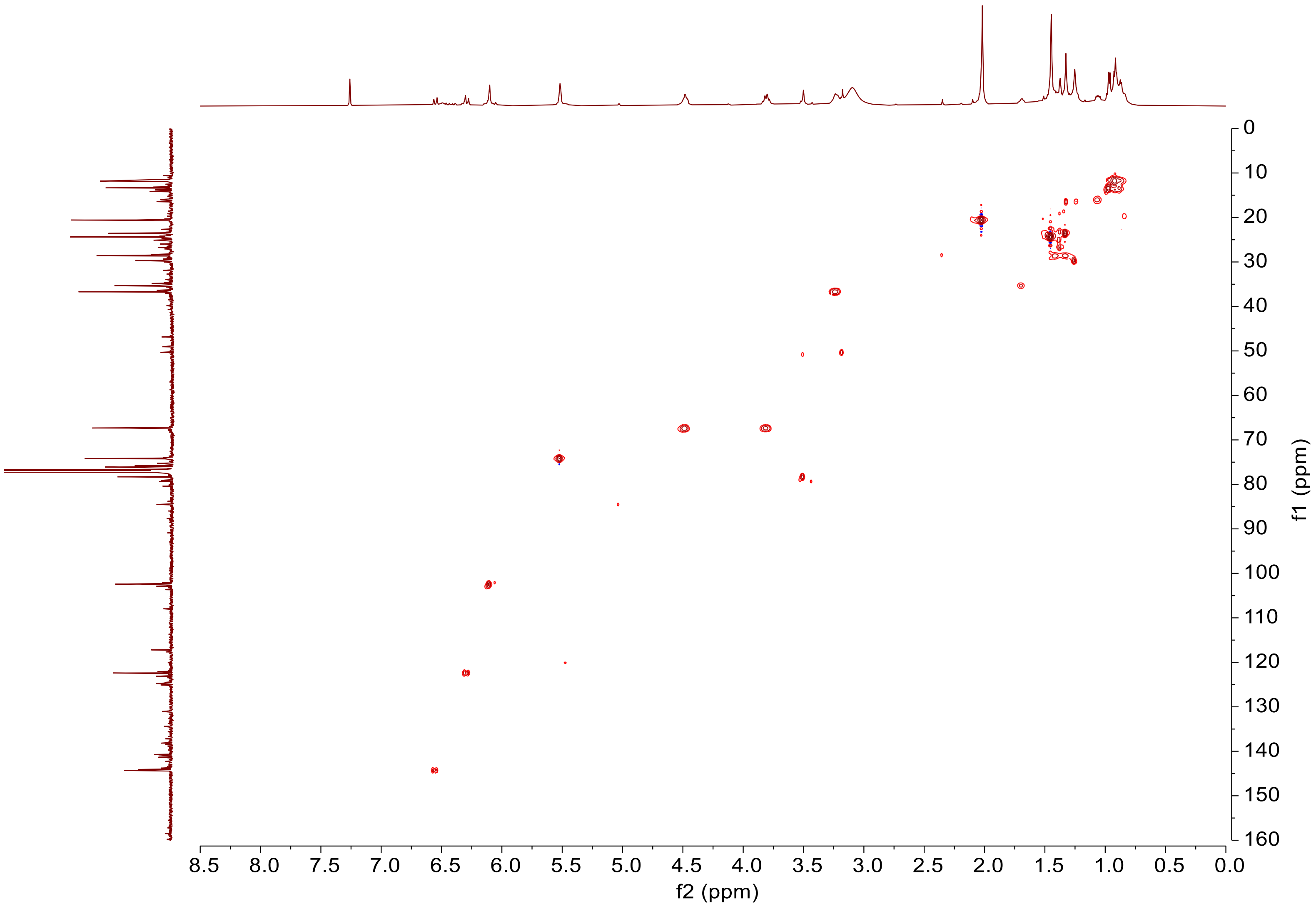

Figure S5 HMBC spectrum of **1**

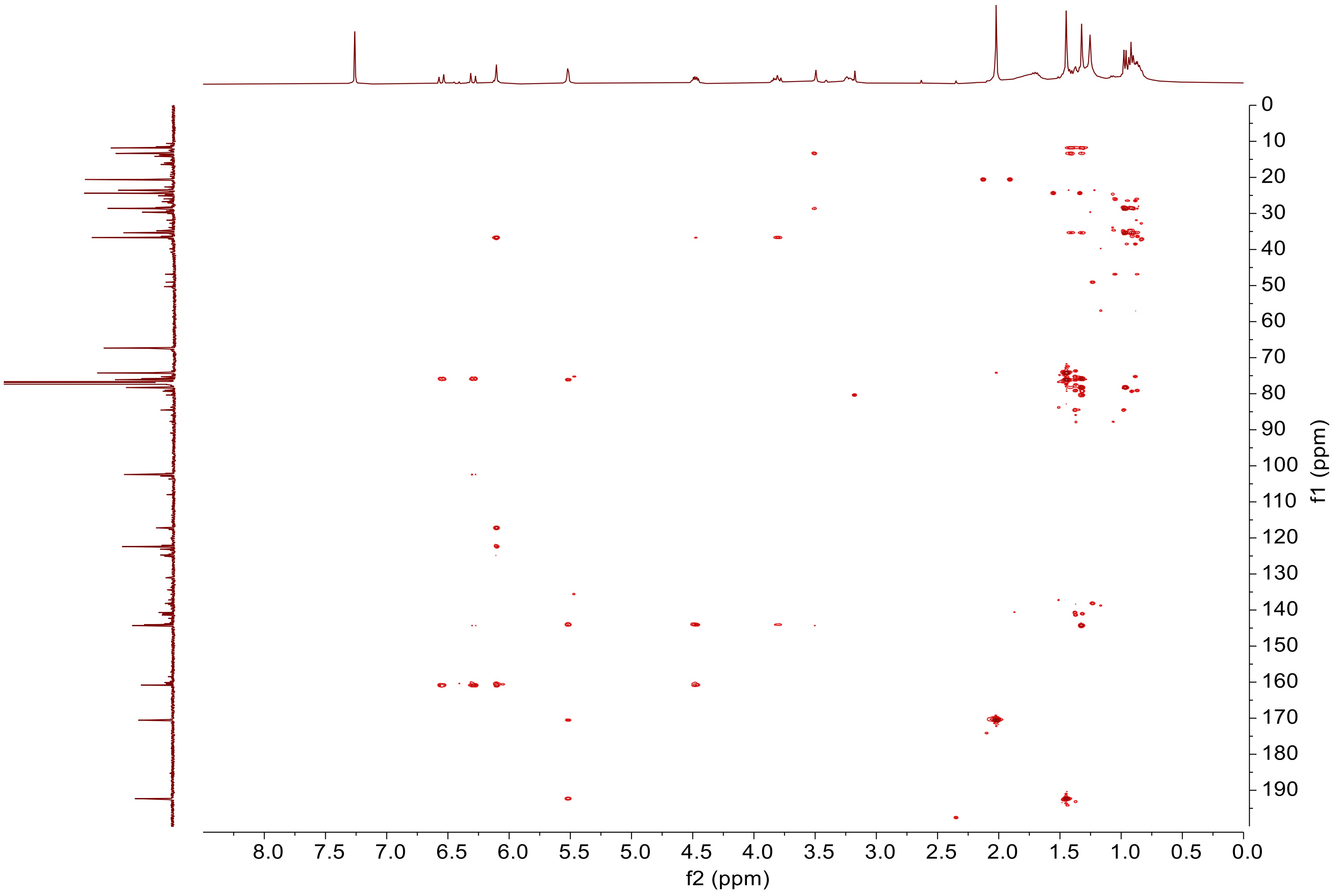

Figure S6 NOESY spectrum of **1**

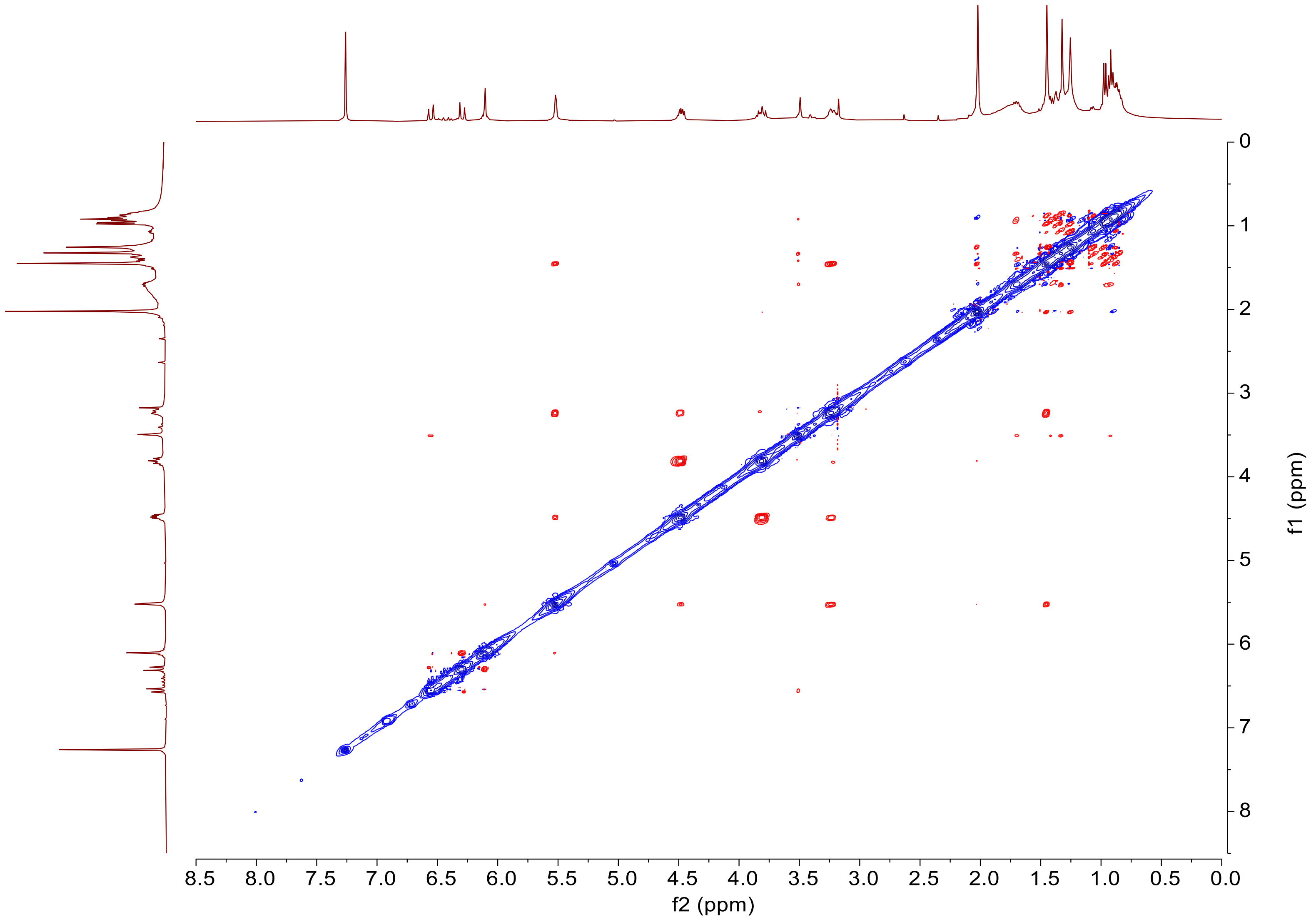

Figure S7 ¹H NMR spectrum of **2** ($CDCl_3$,600 MHz)

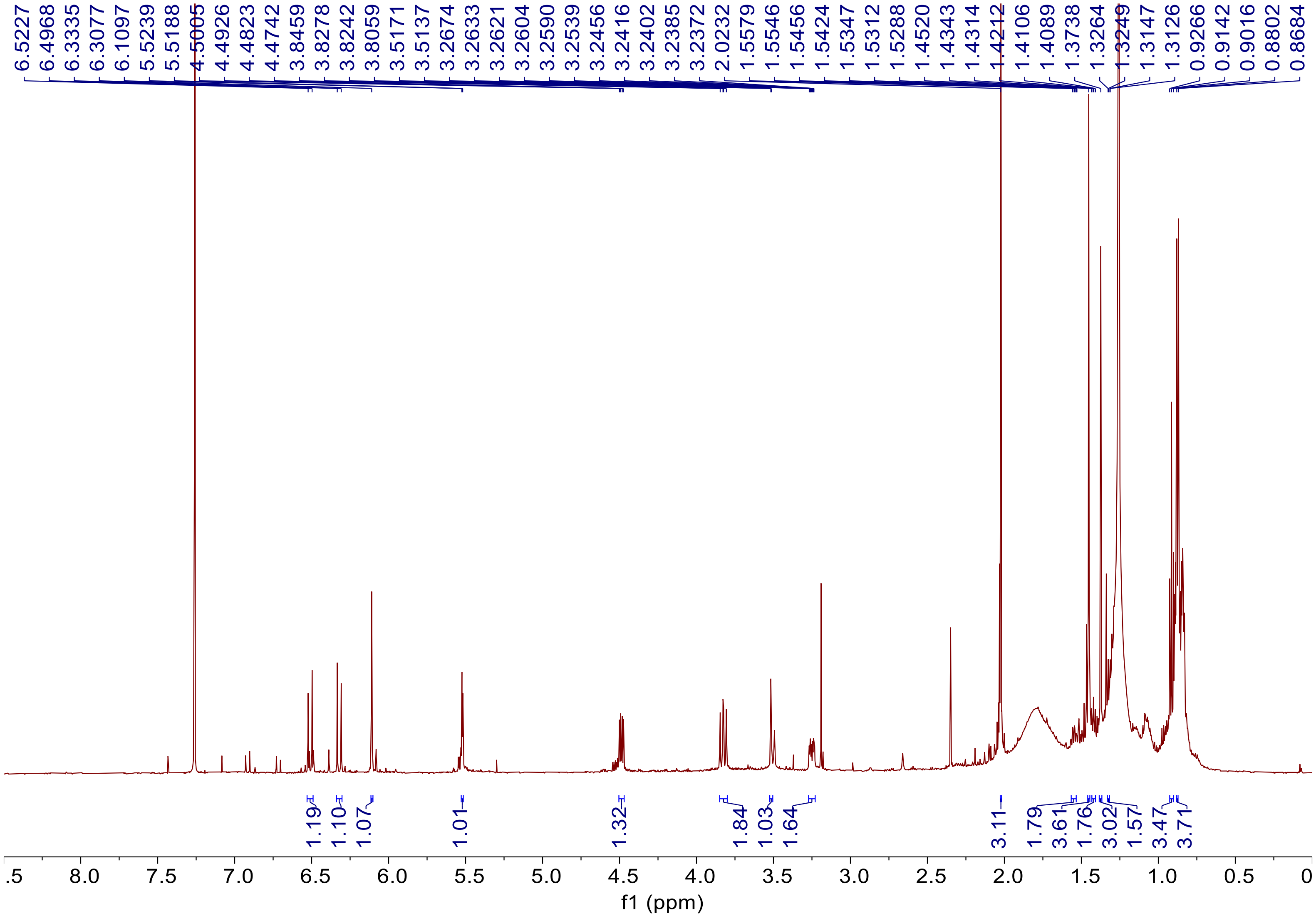

Figure S8 ^{13}C NMR and DEPT spectrum of **2** ($CDCl_3$, 150 MHz)

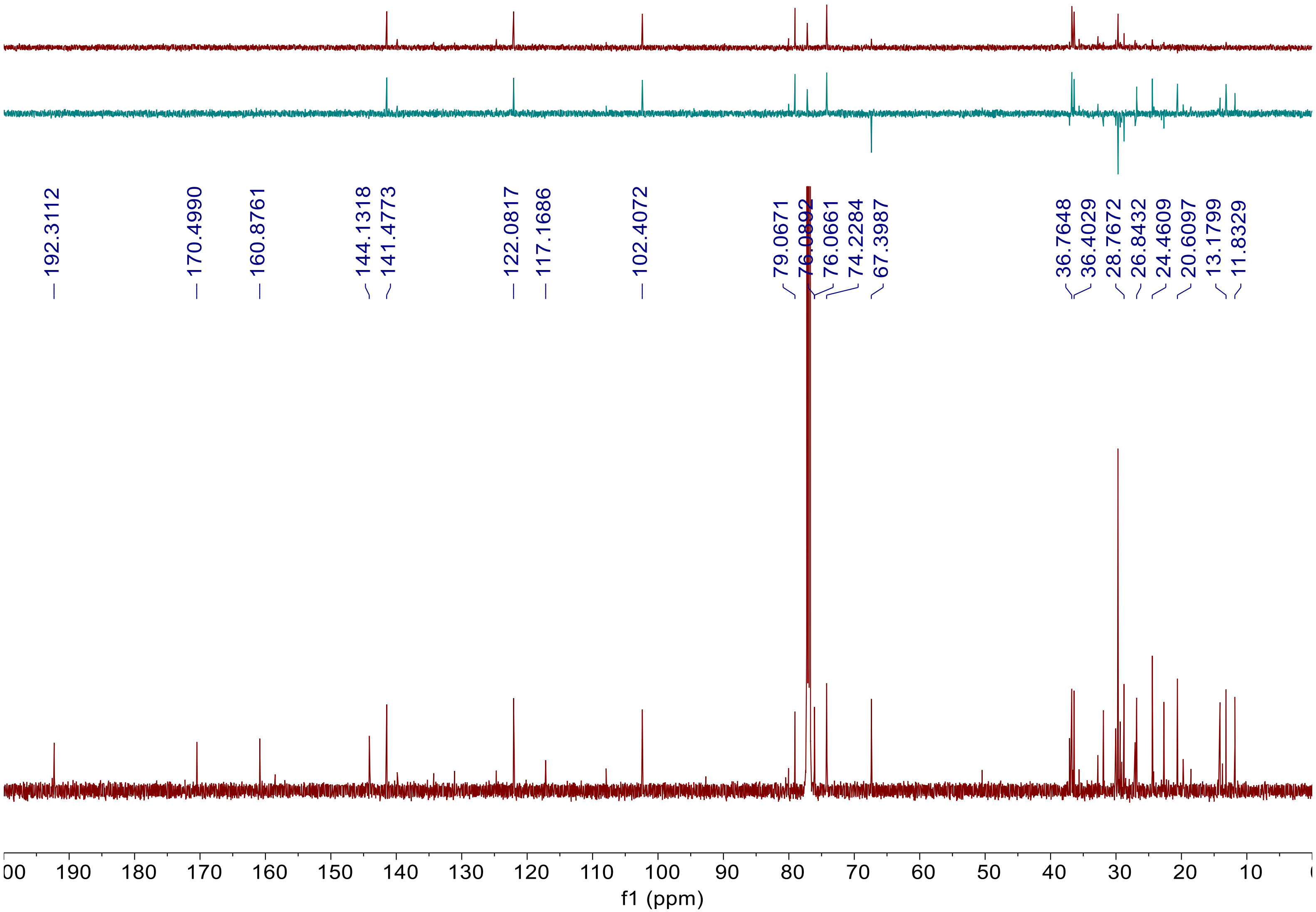

Figure S9 COSY spectrum of **2**

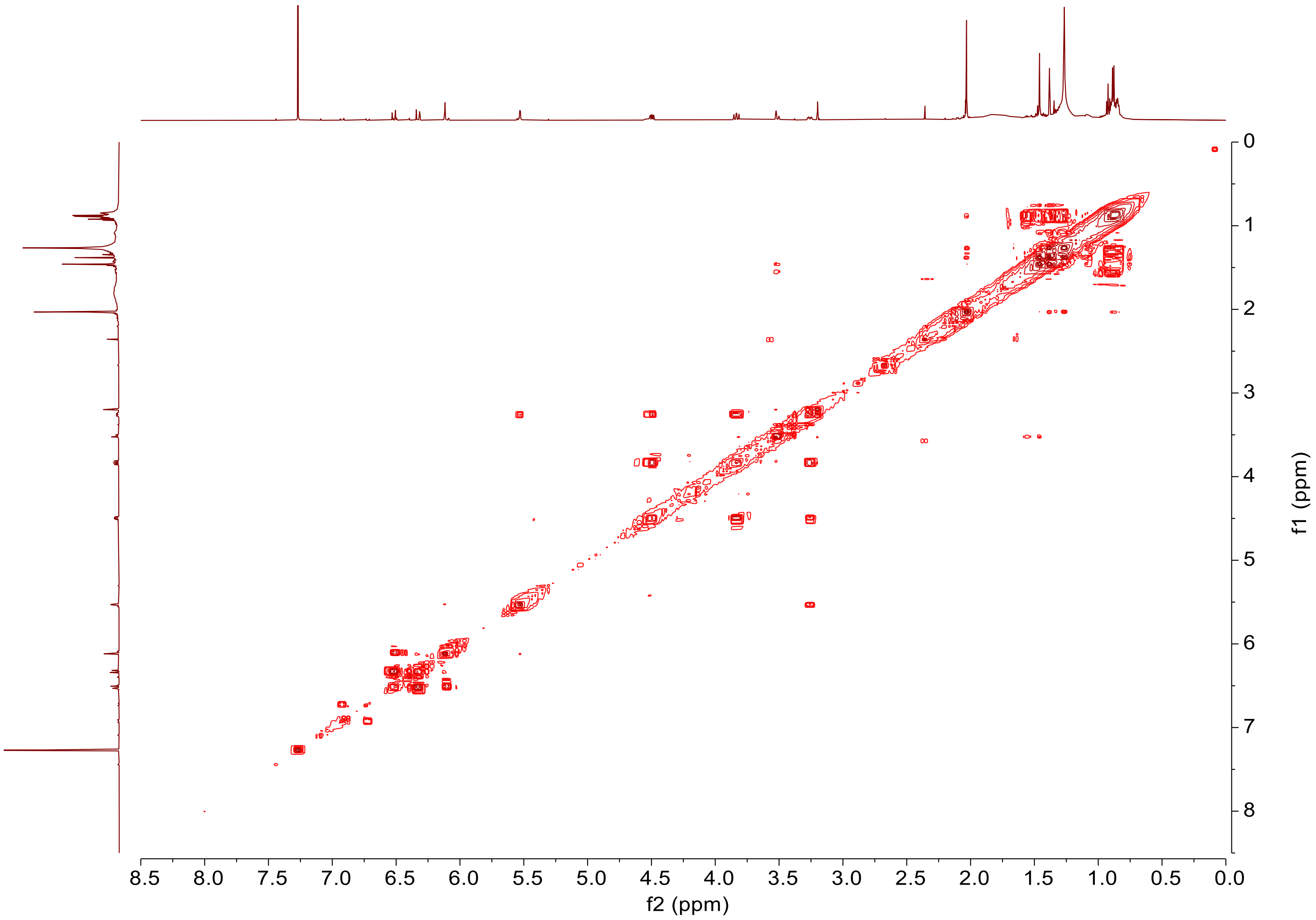

Figure S10 HSQC spectrum of **2**

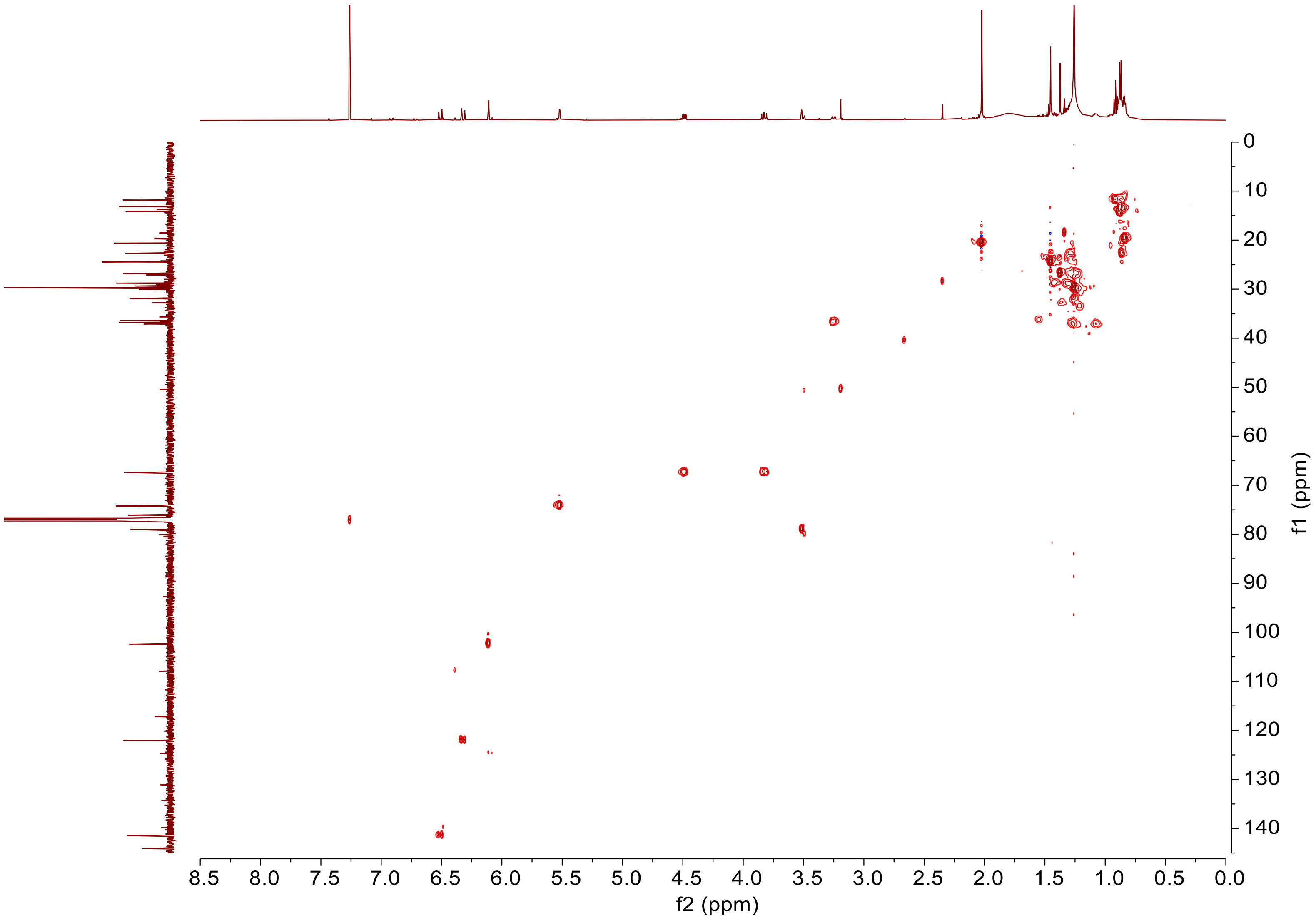

Figure S11 HMBC spectrum of **2**

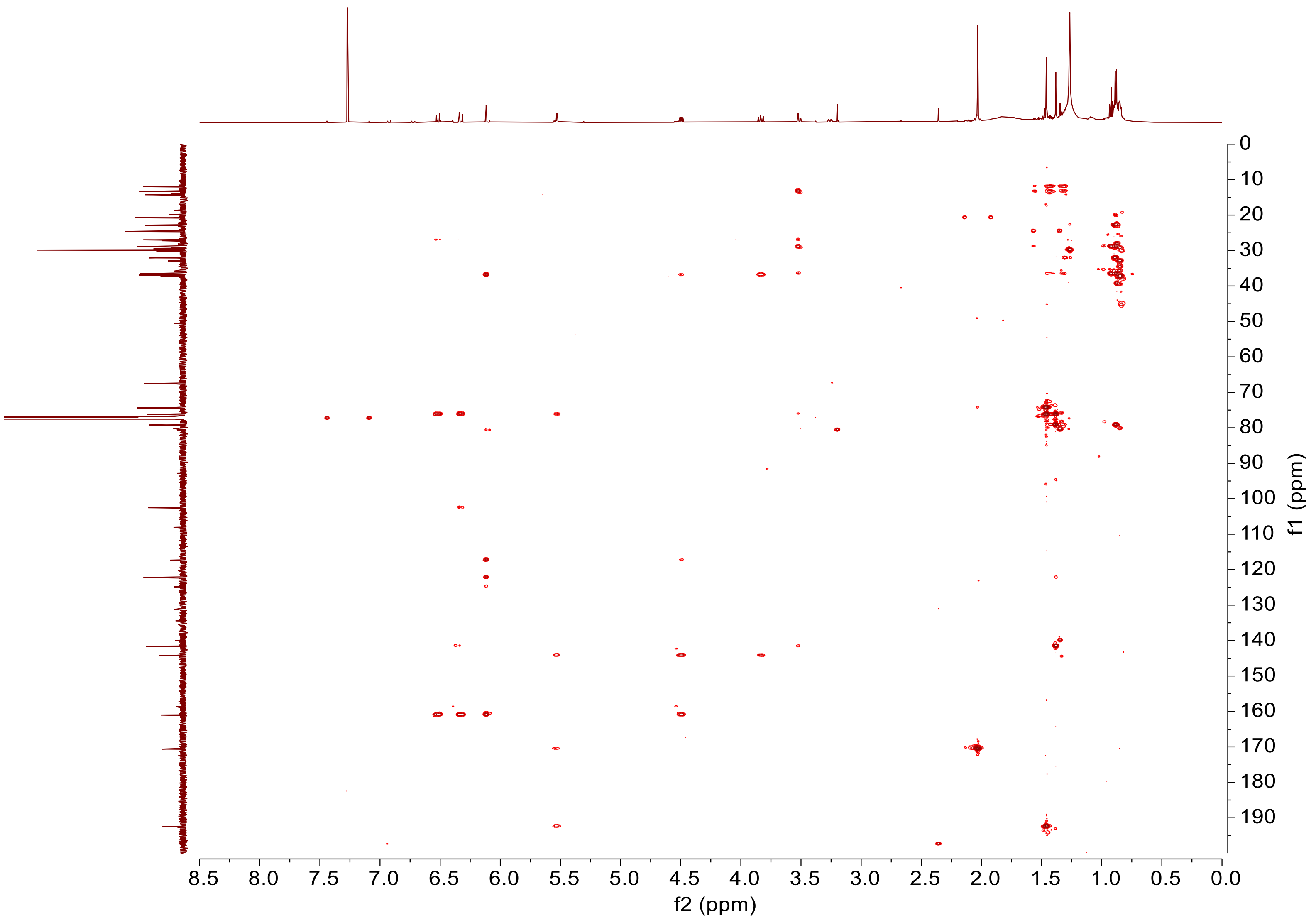

Figure S12 NOESY spectrum of **2**

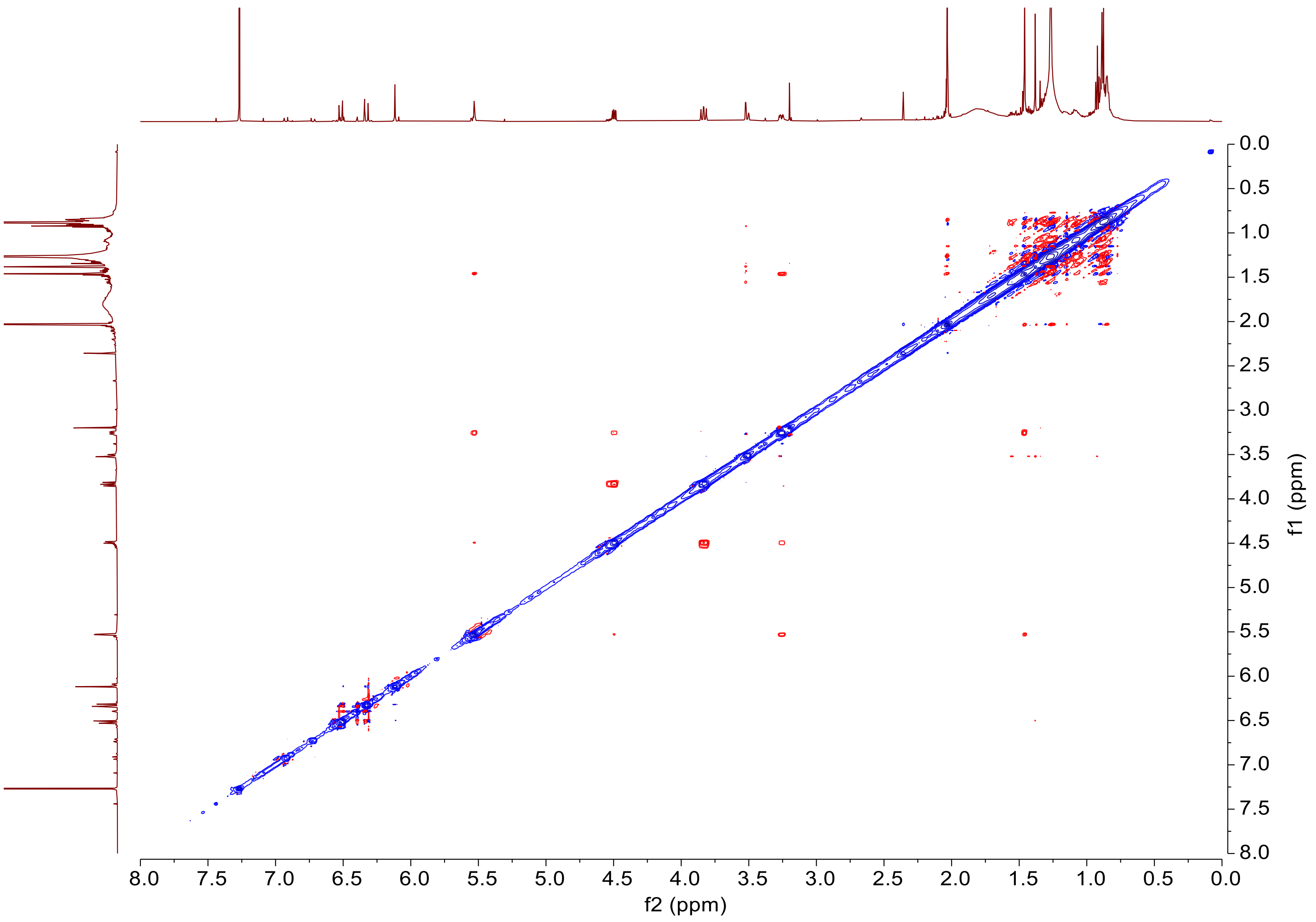

Figure S13 HRESIMS spectrum of **1**

Mass Spectrum SmartFormula Report

Analysis Info

Analysis Name	D:\Data\g4\PSE_000030.d	7/9/2020 1:46:49 PM
Method	broadband first signal	Operator: YU HSIAO-CHING
Sample Name	PS-E	Instrument: BRUKER FT-MS solariX
Comment	ESI Positive	

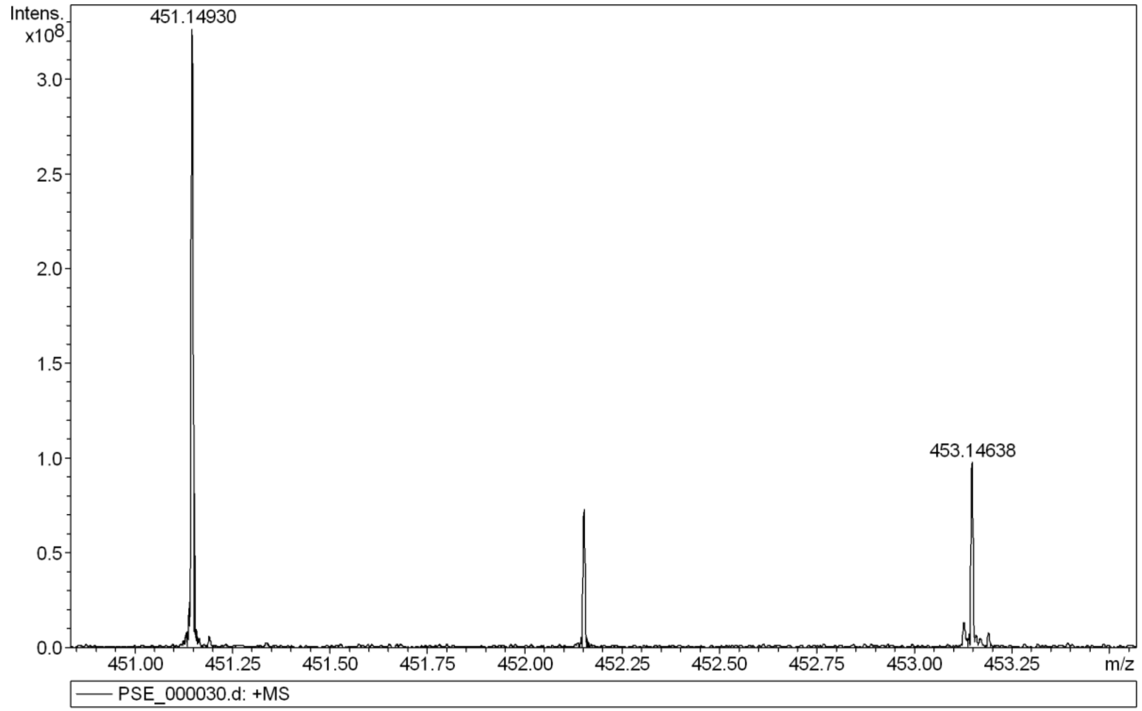

Meas. m/z	#	Formula	Score	m/z	err [mDa]	err [ppm]	mSigma	rdb	e⁻ Conf	N-Rule
451.14930	1	C 21 H 29 Cl Na O 7	100.00	451.14940	0.10	0.23	27.0	6.5	even	ok

Figure S14 HRESIMS spectrum of **2**

Mass Spectrum SmartFormula Report

Analysis Info

Analysis Name	D:\Data\g4\PSF_000005.d	7/9/2020 1:57:57 PM
Method	broadband first signal	Operator: YU HSIAO-CHING
Sample Name	PS-F	Instrument: BRUKER FT-MS solariX
Comment	ESI Positive	

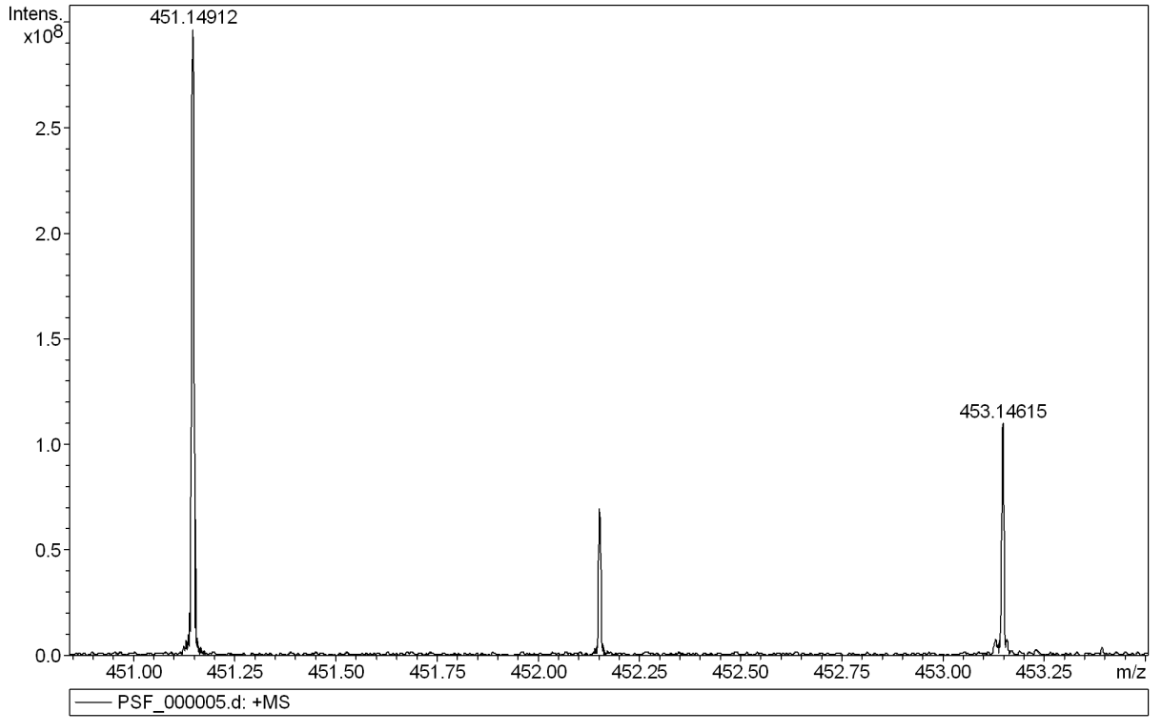

Meas. m/z	#	Formula	Score	m/z	err [mDa]	err [ppm]	mSigma	rdb	e⁻ Conf	N-Rule
451.14912	1	C 21 H 29 Cl Na O 7	100.00	451.14940	0.29	0.63	4.8	6.5	even	ok

Table S1 the ^{1}H and ^{13}C data of the sidechain of compounds **1–4**

	**1**		**3**		**2**		**4**	
	^{1}H	^{13}C	^{1}H	^{13}C	^{1}H	^{13}C	^{1}H	^{13}C
9	6.30, d (15.5)	112.6	6.24 d, (15.5)	122.6	6.32, d (15.5)	112.2	6.30, d (15.6)	122.5
10	6.55, d (15.5)	144.5	6.51 d, (15.5)	144.8	6.51, d (15.5)	141.6	6.51, d (15.6)	141.3
11		76.0		76.1		76.2		76.2
12	3.49, brs	78.5	3.47, brs	78.6	3.52, d (2.0)	79.2	3.50, d (1.5)	79.4
13	1.70, m	35.5	1.69, m	35.4	1.55, m	36.6	1.54, m	35.7
14	1.33, m<br>1.41, m	28.8	1.32, m<br>1.40, m	28.8	1.31, m<br>1.42, m	28.9	1.31, m<br>1.40, m	28.9
15	0.92, t (7.0)	12.0	0.90, t (7.3)	12.0	0.91, t (7.4)	12.0	0.91, t (7.4)	12.0
16	0.97, d (6.8)	13.5	0.98, d (6.7)	13.5	0.88, d (7.0)	13.3	0.87, d (7.0)	13.4
17	1.32, s	23.7	1.30, s	23.7	1.37, s	27.0	1.37, s	26.7

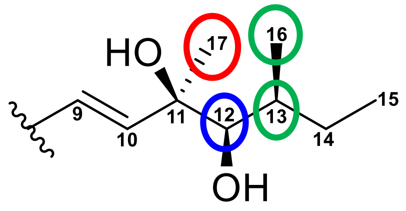

1 & **3**

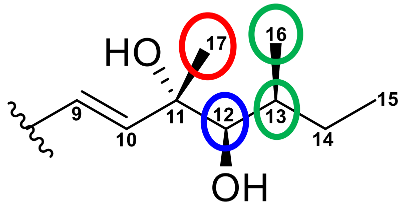

2 & **4**

Figure S15 IR spectrum of **1**

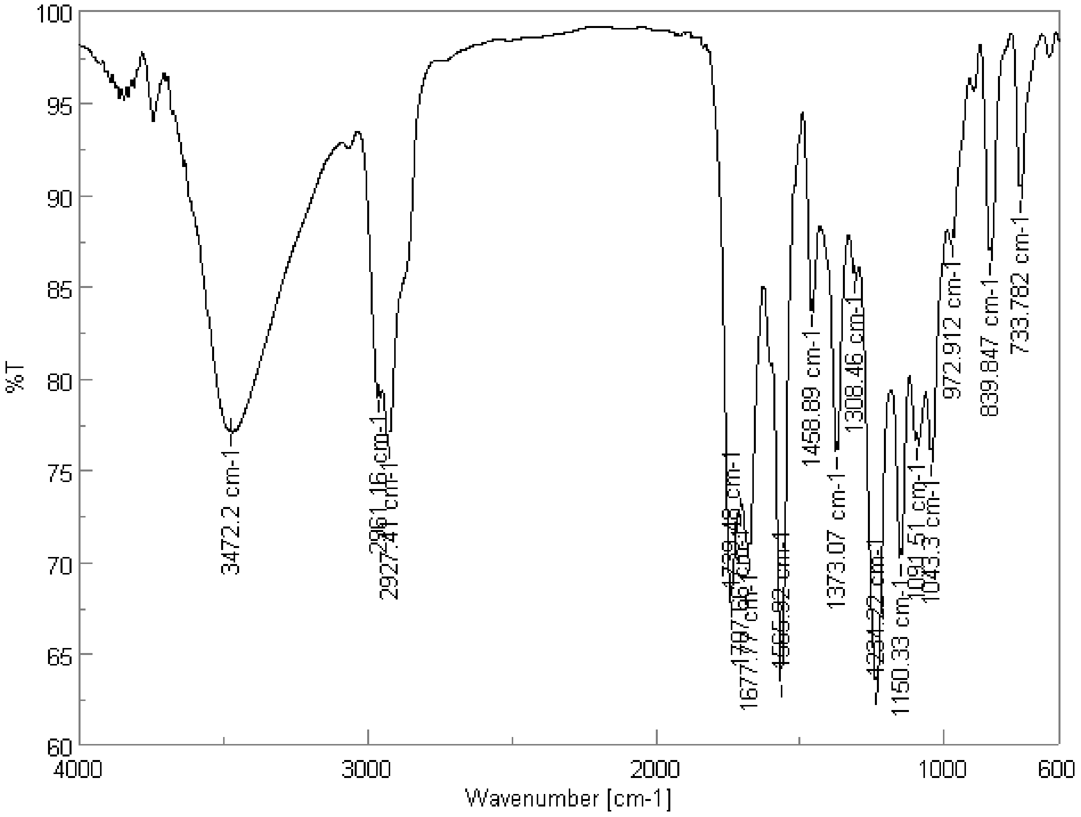

Figure S16 UV spectrum of **1**

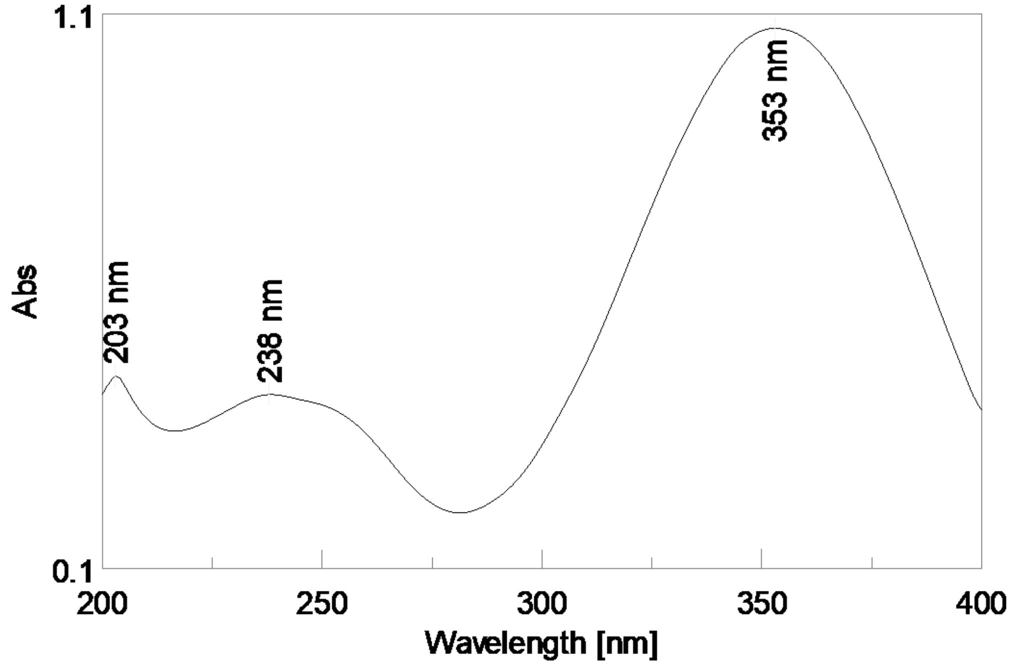

Figure S17 IR spectrum of **2**

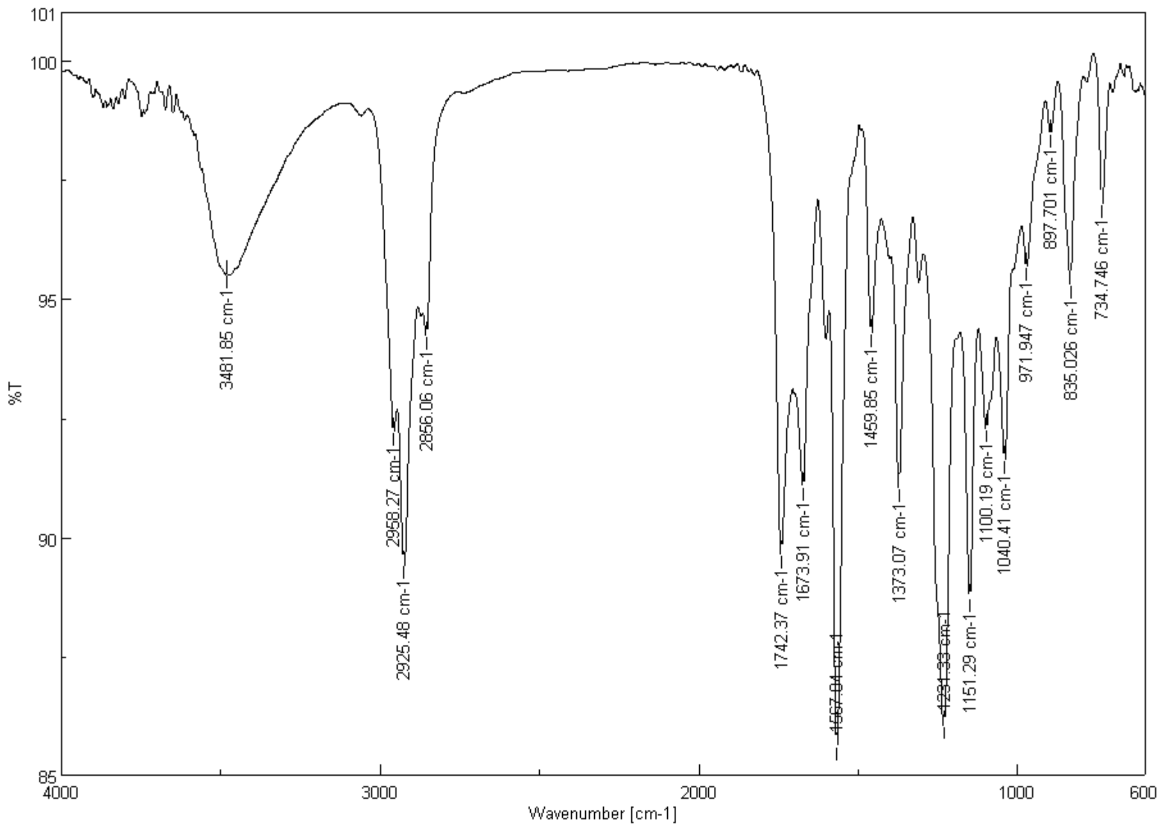

Figure S18 UV spectrum of **2**

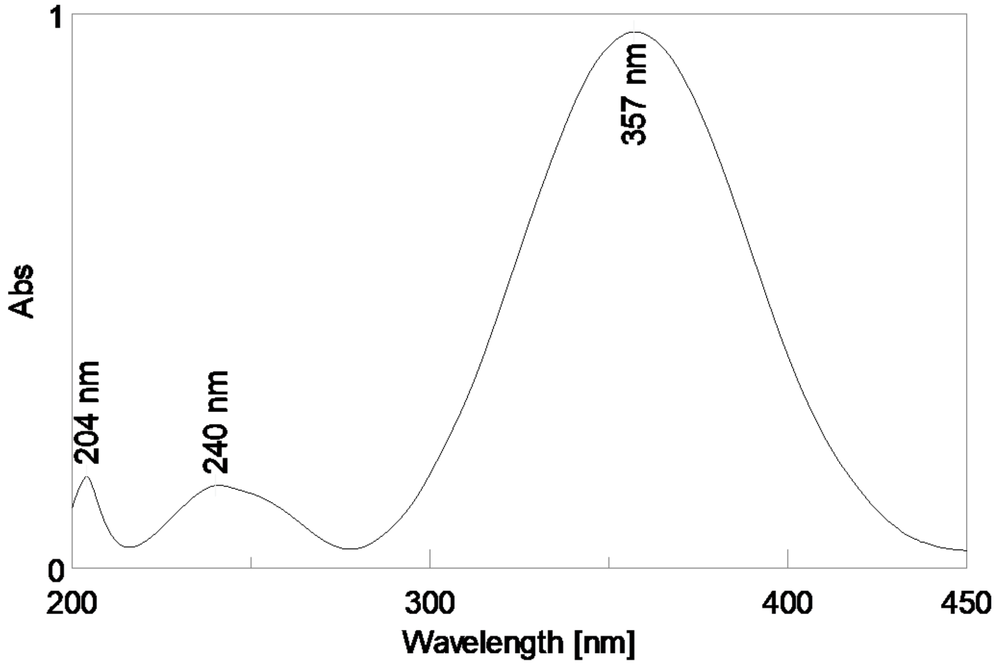

Supplement: Supplementary file 1 [file marinedrugs-19-00529-s001.zip › marinedrugs-1348918-supplementary.pdf]
